# Addressing burning questions on axon regeneration

**DOI:** 10.7554/eLife.101093

**Published:** 2024-08-22

**Authors:** Diána Kaszás, Balázs Enyedi

**Affiliations:** 1 Department of Physiology, Faculty of Medicine, Semmelweis University Budapest Hungary

**Keywords:** wound healing, sensory neuron, reactive oxygen species, burn, epithelial migration, Zebrafish

## Abstract

Regeneration of sensory axons after a burn injury depends on early keratinocyte responses regulated by the wound microenvironment.

**Related research article** Fister AM, Horn A, Lasarev M, Huttenlocher A. 2024. Damage-induced basal epithelial cell migration modulates the spatial organization of redox signaling and sensory neuron regeneration. *eLife* **13**:RP94995. doi: 10.7554/eLife.94995.

In the skin, an extensive network of sensory axons that extend from cell bodies in the spine to allow us to perceive touch, temperature, pain and other stimuli – a process essential for safely navigating our environment. When these axons are damaged through skin injuries, such as cuts or burns, regenerative mechanisms are triggered to restore lost sensations and tissue function.

This process starts with Wallerian degeneration, named after the 19th-century neurophysiologist Augustus Volney Waller (Waller and Owen, 1850). Axon segments that have been separated from their cell bodies experience a rapid influx of calcium, which contributes to the disintegration of the axonal membrane and the myelin sheath that protects it (Geden and Deshmukh, 2016). Immune cells are then recruited to the site to dispose of the damaged cell fragments, clearing the way for new axons to regrow under the influence of various pro-regenerative pathways (Mahar and Cavalli, 2018). The immediate environment surrounding the damaged cell – in particular certain biochemical signals from neighboring skin and nerve cells – plays an important role throughout this process (Avraham et al., 2021). For instance, the reactive oxygen species (ROS) formed after epithelial damage can impact axonal regrowth, either promoting or hindering this process depending on the context (Rieger and Sagasti, 2011; Cobb and Cole, 2015).

Sensory axons have the potential to fully recover after a mechanical injury, such as a cut. However, burn injuries present significant challenges to both skin and neuronal healing, with sensory axons often not fully recovering after this type of wound (Tirado-Esteban et al., 2020). Despite decades of research into the regenerative capacity of sensory and peripheral neurons, a full understanding of this process – and why it is impaired during burn recovery – has remained elusive. Now, in eLife, Anna Huttenlocher and colleagues at the University of Wisconsin-Madison – including Alexandra Fister as first author – report new insights into how the local microenvironment of burnt tissue can influence axon regeneration (Figure 1; Fister et al., 2024).

**Figure 1.**
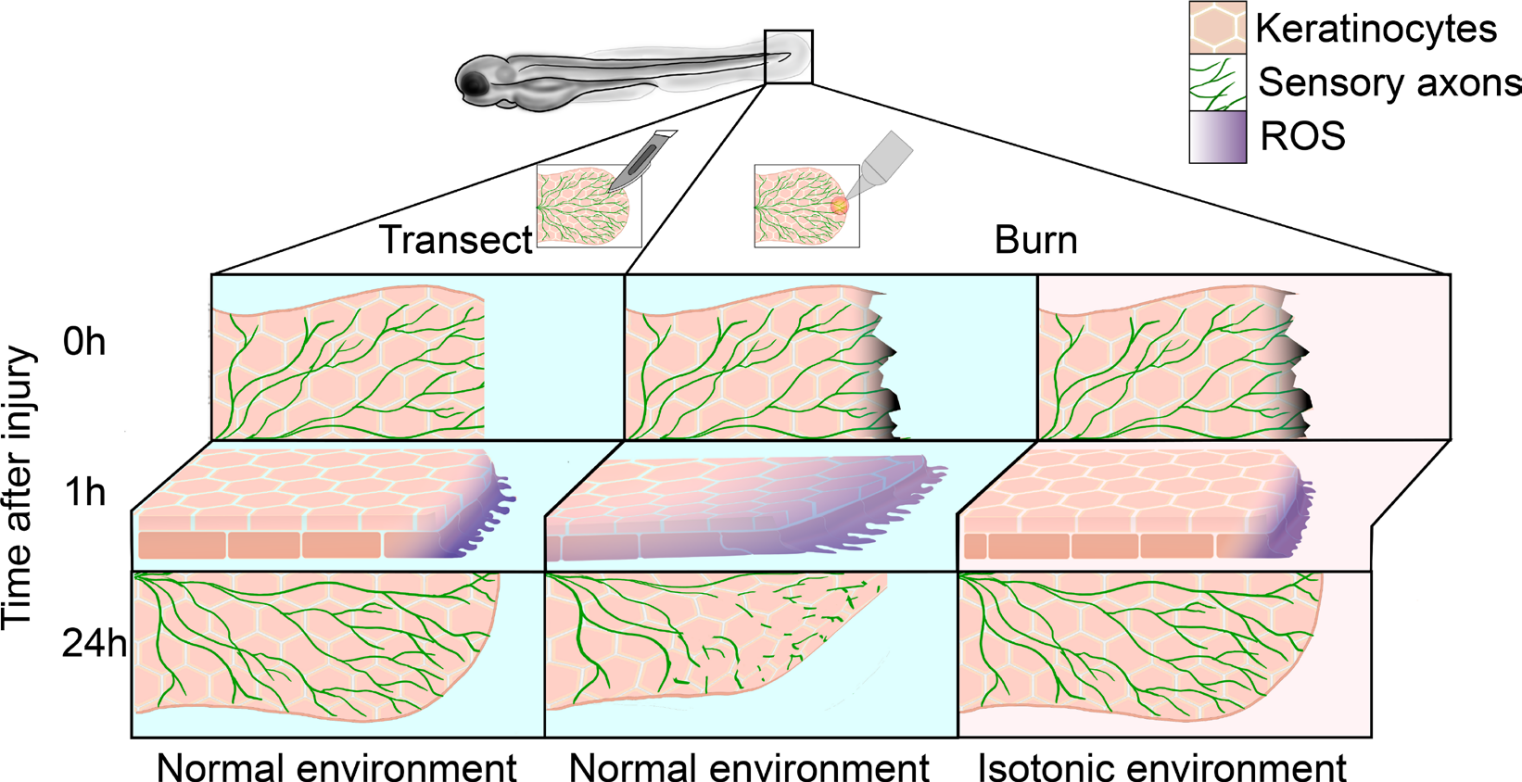
Regeneration of sensory neurons following a tail transection or burn. In normal freshwater medium (blue), transecting the tail fin of a zebrafish larva (left) damages keratinocytes (pink) and sensory axons (green), leading to the production of reactive oxygen species (ROS, purple) at the site of injury within the first hour. After 24hours, sensory axons and keratinocytes have regenerated. In the same conditions (center), burning the tail fin increases the migration of keratinocytes and results in ROS production extending further from the wound edge. There is also impaired sensory axon regeneration and wound healing within the first 24hours. Administering a burn injury in isotonic media (pink, right) rescues both the enhanced keratinocyte migration and the delayed axon regeneration observed in the normal environment.

Capitalizing on a burn wound assay they had previously established in zebrafish larvae, the team first compared how well sensory neurons regrow in these animals after a part of their tails was either burned or amputated (LeBert et al., 2018). This showed that axon healing was delayed by 72hours following a burn. The ability of the wounded area to recover touch sensitivity after a burn was also impaired, which correlated with morphological alterations in the axonal network. In line with previous findings, increased levels of calcium were also found in damaged and fragmented axons after a burn injury.

Elegant experiments showed that excising burnt tissue 5–15minutes after a burn injury led to healing proceeding at a normal pace. Performing the same manipulation six hours after the burn, however, did not accelerate the regeneration process, suggesting that the microenvironment surrounding the damaged tissue contributes to delayed healing.

After a cut, the cells of the deep skin layer (known as basal keratinocytes) collectively migrate between the relatively stationary superficial skin cells and the mesenchymal cells of the connective tissue to facilitate wound closure (Gault et al., 2014). Sensory axons run between the external keratinocyte layer and are also enveloped within the basal layer (O’Brien et al., 2012). To evaluate the impact of the environment on sensory axons, Fister et al. tracked the movement of axons and skin cells using high-resolution live imaging in a series of transgenic zebrafish lines. After a burn, basal cells migrated in an enhanced and uncoordinated manner, dragging keratinocytes in the superficial layers toward the wound and displacing sensory axons from their cell bodies. This early movement was shown to begin at the onset of axonal damage, leading Fister et al. to investigate whether the two events are causally linked.

Two separate methods were used to examine the impact of blocking this migration after a burn. Chemically inhibiting a protein complex required for cell migration slowed early keratinocyte migration but did not change axon regeneration. However, replacing the hypotonic freshwater in which zebrafish normally live with an isotonic saline solution while administering a burn facilitated axon regeneration and rapid recovery of tail fin touch sensitivity. Indeed, previous reports on mechanical injuries had shown that initial wound closure and basal cell migration are triggered by exposure to hypotonic freshwater, which, unlike isotonic saline, encourages water to enter cells, resulting in osmotic cell swelling (Gault et al., 2014). However, improvements in regeneration and sensitivity were not observed if the larvae were only exposed to isotonic saline one hour after the burn injury, supporting the idea that the local mechanisms influencing axon regeneration take place shortly after burning occurs.

To identify the environmental signals involved, Fister et al. next tracked ROS production in recovering wounds. Compared to cuts, increased ROS signals were detected in burns further from the wound margin. Preventing basal cell migration either chemically or via exposure to isotonic saline blocked enhanced ROS production deep inside the burn injury, but not at its edges. Taken together, this suggests that the enhanced keratinocyte migration observed after a burn disrupts how and where ROS are produced during early recovery. Blocking production of ROS either chemically or genetically proved fatal for the larvae, and therefore further experiments are required to establish whether ROS signals directly delay axon regeneration in burns.

The findings of Fister et al. identify novel links between the local microenvironment, ROS production and the regenerative capacity of peripheral sensory axons in response to burn wounds. Overall, the experiments indicate that burns lead to enhanced and uncoordinated basal keratinocyte migration that displaces sensory axons, which is associated with the start of axonal damage. At the same time, it remains unclear whether exposing the burn to isotonic conditions rescues axon regeneration by blocking keratinocyte migration, or through further mechanisms. Notably, exposing wounded or burnt tissue to the normal hypotonic environment of zebrafish leads to osmotic cell swelling, which is completely blocked under isotonic conditions. Investigating whether any signaling pathways and cellular responses related to swelling contribute to the beneficial effects of isotonic saline could be worthwhile in future studies.
